# Plasma lncRNA signature of upregulated ATP2B1-AS1 and downregulated RPL21P28 correlates with diagnosis and cognitive severity in Alzheimer’s disease

**DOI:** 10.3389/fnagi.2026.1739935

**Published:** 2026-06-03

**Authors:** Atefe Yadollahi Khales, Kamran Ghaedi, Fariba Esmaeili, Maryam Noorbakhshnia, Masoud Etemadifar

**Affiliations:** 1 Department of Plant and Animal Biology, Faculty of Biological Science and Technology, University of Isfahan, Isfahan, Iran; 2 Department of Biology, Faculty of Biological Science and Technology, University of Isfahan, Isfahan, Iran; 3 Department of Neurology, Isfahan University of Medical Sciences, Isfahan, Iran

**Keywords:** Alzheimer’s disease, ATP2B1-AS1, lncRNAs, non-invasive biomarkers, RPL21P28

## Abstract

**Background:**

The non-invasive biomarkers have considerable effects in determination and treatment of Alzheimer’s disease (AD). The specificity and stability of the circulating long non-coding RNAs (lncRNAs) have made them suitable options in disease management. The aim of this study was discovering and clinically confirming a new plasma lncRNA signature and the underlying regulatory mechanisms.

**Methods:**

In a two-stage research, the preliminary bioinformatic screen of public transcriptomic data (GEO: GSE63060) found candidate lncRNAs. Then the level of the major candidates, ATP2B1-AS1 and RPL21P28, were validated by Real-Time qPCR in plasma of 25 AD patients and 25 healthy controls. The diagnostic performance was appraised by the Receiver Operating Characteristic (ROC) curve analysis and the presumed functions were described by a competing endogenous RNA (ceRNA) network.

**Results:**

Our study established concurrent ATP2B1-AS1 upregulation and RPL21P28 downregulation in Alzheimer’s patient plasma (*p* < 0.001). This highly discriminatory two-lncRNA signature produced an area under the curve (AUC) of 0.81 for ATP2B1-AS1 and 0.83 for RPL21P28. Based on MMSE scores, the expressions of both lncRNAs were significantly correlated with the level of cognitive impairment. These lncRNAs were also showed a correlation to important regulation mechanisms by our ceRNA network analysis, with RPL2B1P28 linked to synaptic functional genes and ATP2B1-AS1 to neurodevelopment.

**Conclusion:**

ATP2B1-AS1 and RPL21P28 plasma levels present specific AD biomarker signature. These lncRNAs show great potentials in designing a non-invasive blood test that can be used for early diagnosis and disease follow-up. It also establishes new areas for intervention therapy research.

## 1 Introduction

Alzheimer’s disease (AD) is the single most prevalent neurodegenerative disease and the leading cause of dementia around the world, which not only affects patients themselves and their relatives but also poses a heavy burden on the healthcare system (Alzheimer’s Association, 2023). AD, a disease of progressive memory loss and other declining cognitive function, is estimated to impact over 250 million people worldwide in 2050 (GBD 2019 Dementia Forecasting Collaborators, 2022).

Clinical investigation and neuropsychological testing, and in particular biomarkers obtained from CSF examination or high-end neuroaxis imaging (e.g., PET scans), are essential for an early diagnosis of AD (Jack et al., 2018). Although these methods can be useful, they can be costly, invasive, and not highly sensitive for disease detection at the early/asymptomatic stage (Hampel et al., 2023).

The increased intracellular levels of neurofibrillary tangles (NFTs) of hyperphosphorylated tau protein and extracellular levels of amyloid-beta (Aβ) plaques are established as the major molecular hallmarks of AD (Sadeghi et al., 2025). The amyloid cascade hypothesis (that the accumulation of Aβ initiates a subsequent cascade of events of tau pathology, synaptic impairment, and neuroinflammation) has been the dominant theory of AD for several decades (Gulisano et al., 2018).

The poor results of studies on Aβ-targeting drugs in clinical experiments, especially the lack of effectiveness of gantenerumab, have questioned the validity of this model (Ali et al., 2025). The recent antibodies like lecanemab, with a moderate level of efficacy, indicate the complexity of AD and highlight the need to focus on the upstream regulatory mechanisms that are directly related to disease pathogenesis (Fertan et al., 2025).

The non-coding regions of the genome have been proven to play significant parts in biomedical studies. Today, these long non-coding RNAs (lncRNAs) are noted as the chief regulators of gene expression (Nie, 2012). They can act as molecular scaffolds, modulate the activity of proteins, and direct chromatin-modifying complexes (Kopp and Mendell, 2018).

Due to the high stability and tissue specificity of RNAs, these biomolecules are suitable options to be used as biomarkers for non-invasive liquid biopsy (Wylezinski et al., 2020). Dysregulation of lncRNA through synthesis of Aβ, tau phosphorylation, and neuroinflammation plays a vital role in the pathogenesis of neurodegenerative disorders, including AD (Diling et al., 2020).

lncRNAs can exert their regulatory effect via the competing endogenous RNA (ceRNA) network hypothesis (Salmena et al., 2011). By binding to specific miRNAs, lncRNAs can act as “microRNA (miRNA) sponges,” preventing them from interacting with their messenger RNA (mRNA) targets (Tay et al., 2014). A modified, sophisticated signaling network is provided by this extremely complex regulatory crosstalk, where the dysregulation of one lncRNA can affect several protein-coding genes. While it is now clear that particular lncRNAs such as BACE1-AS regulate Aβ synthesis in brain tissues (Faghihi et al., 2008), the exact profile of lncRNAs in AD and their connection with ceRNA networks have not been fully investigated in systematic review studies (Cai et al., 2022).

Therefore, the current research designed and executed a systematic, two-stage validation study. We aimed to apply a high-stringency bioinformatic screen of large-scale transcriptomic data to find new lncRNA dysregulations in the blood samples of AD patients; verify the mRNA expression of the main candidates, ATP2B1-AS1 and RPL21P28, as blood-based markers of AD; and investigate their possible functions in the pathogenesis of AD by outlining their respective ceRNA regulatory networks.

## 2 Materials and methods

### 2.1 Study design and ethical approval

This discovery-to-validation research was carried out in two stages. The early discovery stage included a bioinformatic analysis on available transcriptomic data for identifying and enlisting the candidate lncRNAs. The following validation stage included an experimental quantitation of the selected candidates in a clinically verified case-control sample. This study was approved by the Institutional Review Board of the University of Isfahan, Iran (Ethical Code: [IR.UI.REC.1401.127]). All procedures were carried out in accordance with the ideals of the Declaration of Helsinki. All participants, or their legal guardians, signed written informed consent forms before study inclusion.

### 2.2 Phase 1: bioinformatic discovery and candidate selection

#### 2.2.1 Data acquisition and preprocessing

In order to select the suitable lncRNAs candidates for the study of AD, raw microarray data from four separate experiments (GEO accession numbers: GSE4757, GSE5281, GSE28146, and GSE48350), which were all created by the Affymetrix Human Genome U133 Plus 2.0 Array platform, were downloaded from the NCBI Gene Expression Omnibus (GEO) repository. Pre-processing of the raw data files (CEL) was executed by the affy R package version 4.1.2. Background correcting, quantile normalizing, and log2 transforming were all carried out by the Robust Multi-array Average (RMA) procedure (Irizarry et al., 2003). For variability control of the inter-study, the RMA-regularized data matrices were integrated according to shared probe IDs, and the ComBat procedure of the sva R package eliminated the batch effects (Johnson et al., 2007).

#### 2.2.2 Differential expression analysis

Differential expression analysis between AD patients and healthy controls was performed using linear models implemented in the limma R package (Ritchie et al., 2015). To control for multiple comparisons, *P*-values were adjusted using the Benjamini–Hochberg procedure in order to control the false discovery rate (FDR). Probe sets with an adjusted *P*-value < 0.05 and an absolute log2(fold change) > 0.58 were considered significantly dysregulated.

To identify lncRNA candidates, probe annotations were examined and those corresponding to long non-coding RNAs were selected. Based on the magnitude of differential expression and biological plausibility, two lncRNAs, ATP2B1-AS1, and RPL21P28, were selected as candidate molecules for subsequent experimental validation.

### 2.3 Phase 2: clinical validation cohort and sampling

#### 2.3.1 Participant and clinical recruitment

A total of 25 patients with suspected AD and 25 age-and gender-matched healthy controls were enrolled in this study from the Clinic of Cellular, Molecular and Genetics Research Center in Isfahan, Iran.

#### 2.3.2 Alzheimer’s disease diagnostic criteria

The diagnosis of probable Alzheimer’s disease (AD) was established by an experienced neurologist based on the National Institute on Aging and Alzheimer’s Association (NIA-AA) workgroup criteria. Neuroimaging evidence of neurodegeneration was evaluated using brain magnetic resonance imaging (MRI) to support the clinical diagnosis. In this cohort, CSF biomarkers or amyloid/tau PET imaging were not routinely available; therefore, diagnosis was primarily based on clinical assessment and MRI findings.

Cognitive status was assessed using the Mini-Mental State Examination (MMSE). Patients with AD who had MMSE scores between 10 and 24, corresponding to mild to moderate cognitive impairment, were included in the study. Individuals with severe cognitive impairment or other neurological and psychiatric disorders that could influence cognitive function, such as frontotemporal dementia, Lewy body dementia, vascular dementia, or major depressive disorder, were excluded.

Healthy control participants were recruited from the same geographical region and were screened to confirm the absence of neurological or psychiatric disorders. All control subjects were cognitively normal with MMSE scores > 27. Demographic and clinical characteristics of the study participants are summarized in Table 1.

**TABLE 1:** Demographic and clinical features of study participants.

Characteristic	Patients with AD (*n* = 25)	Healthy controls (*n* = 25)	*P*-value
Age (years, mean ± SD)	74.5 ± 6.2	73.8 ± 5.9	73.8 ± 5.9
Sex (male/female)	14/11	13/12	0.785^b^
MMSE score (mean ± SD)	18.2 ± 4.5	28.9 ± 1.	< 0.0001^a^

AD, Alzheimer’s Disease; SD, Standard Deviation; MMSE, Mini-Mental State Examination.

^a^*P*-value that has been adjusted.

^b^*P*-value calculated by Chi-square test.

The sample size of 25 AD patients and 25 healthy controls was determined based on feasibility and the availability of eligible participants in this single-center study. This sample size was considered adequate for an initial validation of the selected lncRNA biomarkers and for exploratory receiver operating characteristic (ROC) analysis, although it may limit the statistical power to detect smaller effect sizes or to perform subgroup analyses.

#### 2.3.3 Plasma collection and processing

A volume of 10 mL of venous blood was obtained from all participants of the study into EDTA- coated tubes. Samples were processed within an hour of blood collection using a standard two-step centrifugation procedure to acquire platelet-poor plasma. The blood samples were spun at 4°C at 1,500 × g. To separate the sedimented remaining cells, platelets, and large extracellular vesicles, the supernatant (plasma) was carefully transferred to a new sterile tube and spun at 4°C at 16,000 × g for 10 min (Spence, 2004). The final plasma supernatant was aliquoted and stored at −80°C prior to RNA extraction.

### 2.4 RNA extraction and quality control

The miRNeasy Serum/Plasma Kit (Qiagen, Hilden, Germany) was applied for extracting the total RNA, including long and small non-coding RNA species, from 200 μL of plasma according to the manufacturer’s instructions. The extracted RNA eluted into 14 μL of RNase-free water. The purity and concentration of extracted RNA were measured using the Qubit RNA HS (High Sensitivity) Assay Kit on a Qubit 4 Fluorometer (Thermo Fisher Scientific, Waltham, MA, United States), which is suitable for measuring low-concentrated substances.

### 2.5 Reverse transcription and real-time quantitative PCR (RT-qPCR)

The miScript II RT Kit (Qiagen) was applied for synthetizing the complementary DNA (cDNA) from 5 μL of total RNA based on the manufacturer’s instructions. RT-qPCR was carried out on a QuantStudio 6 Flex Real-Time PCR System (Applied Biosystems, Foster City, CA, United States). Reactions were performed three times in a final volume of 10 μL, containing the miScript SYBER Green PCR Kit master mix (Qiagen), 1 μL of the cDNA template, and proper forward and reverse primers. Table 2 displays the sequence of primers. The endogenous reference gene applied to normalized the expression of the test genes was glyceraldehyde-3-phosphate dehydrogenase (GAPDH). The applied thermal cycling order was as follows: the first denaturation cycle at 95°C for 15 min, and then 40 cycles of 94°C for 15 s, 55°C for 30 s, and 70°C for 30 s. The specificity of the PCR-based amplified product was validated using a melt curve analysis at the end of each run.

**TABLE 2:** The applied primers in RT-qPCR analysis.

Target	Primer	Sequence (5’–3’)
ATP2B1-AS1	Forward	GCCCAGAGACCTTGTCAGT
	Reverse	ACTTCCTATTGCCTGTATGCTT
RPL21P28	Forward	CAAAGGGAAAGAGGAGAGG
	Reverse	CCGTTCCCTTGATGTCTAC
GAPDH	Forward	AACAGCCTCAAGATCATCAGC
	Reverse	CCATCACGCCACAGTTTCC

### 2.6 Building ceRNA

In line with bioinformatic anticipations, a ceRNA network was established in order to examine the theoretical regulatory roles of the identified lncRNAs. The StarBase v3.0 database identified miRNAs that have been experimentally confirmed to target ATP2B1-AS1 and RPL21P28 (Yang et al., 2011). Afterward the mRNA targets of the identified miRNAs were anticipated using the Target Scan (version 8.0) and miRDB databases. Only the mRNA targets that both programs predicted were kept for further study. The final tripartite lncRNA-miRNA-mRNA network was determined and visualized using the Cytoscape version 3.9.1 software (Shannon et al., 2003).

### 2.7 Statistical

The GraphPad Prism software (versions 9.0, GraphPad Software, San Diego, CA, United States) was applied for statistical analyses. The 2^–ΔΔ^*^CT^* method was used compute and determine the relative lncRNA expressions, with the mean of the control group functioning as a calibrator (Livak and Schmittgen, 2001). The normal distribution of data was examined using the Shapiro-Wilk test. To define and compare the expression levels between the AD patients and the control group, an unpaired *t*-test (for normally distributed data) or a Mann-Whitney U test (for non-normally distributed data) was accomplished. The diagnostic efficacy of each lncRNA was assessed by the receiver operating characteristic curve analysis and the area underneath the curve calculation. The correlation of the level of lncRNA expressions and the clinical score (MMSE) were analyzed by Spearman’s rank correlation coefficient test. The *P*-value < 0.05 was considered statistically significance.

For the primary comparisons between AD patients and healthy controls in the clinical validation cohort, focus was placed on the two pre-selected lncRNAs (ATP2B1-AS1 and RPL21P28) identified from the bioinformatic discovery phase. Given this focused validation approach, a formal multiple comparison correction (e.g., Bonferroni or FDR) was not applied to the *t*-tests/Mann-Whitney U tests for group comparisons or to the correlation analyses in this phase. However, the potential for type I error due to multiple testing across different analyses is acknowledged as a limitation of the study. The diagnostic performance was assessed using ROC analysis, where AUC values and their corresponding confidence intervals provide an integrated measure of diagnostic accuracy.

Because the clinical validation stage evaluated only two pre-selected lncRNA candidates (ATP2B1-AS1 and RPL21P28) in a hypothesis-driven manner, no additional correction for multiple comparisons was applied in this phase of the analysis.

## 3 Results

### 3.1 Bioinformatic screening of public datasets distinguishes ATP2B1-AS1 and RPL21P28 as the two most dysregulated lncRNAs in the blood samples of AD patients

**FIGURE 1:**
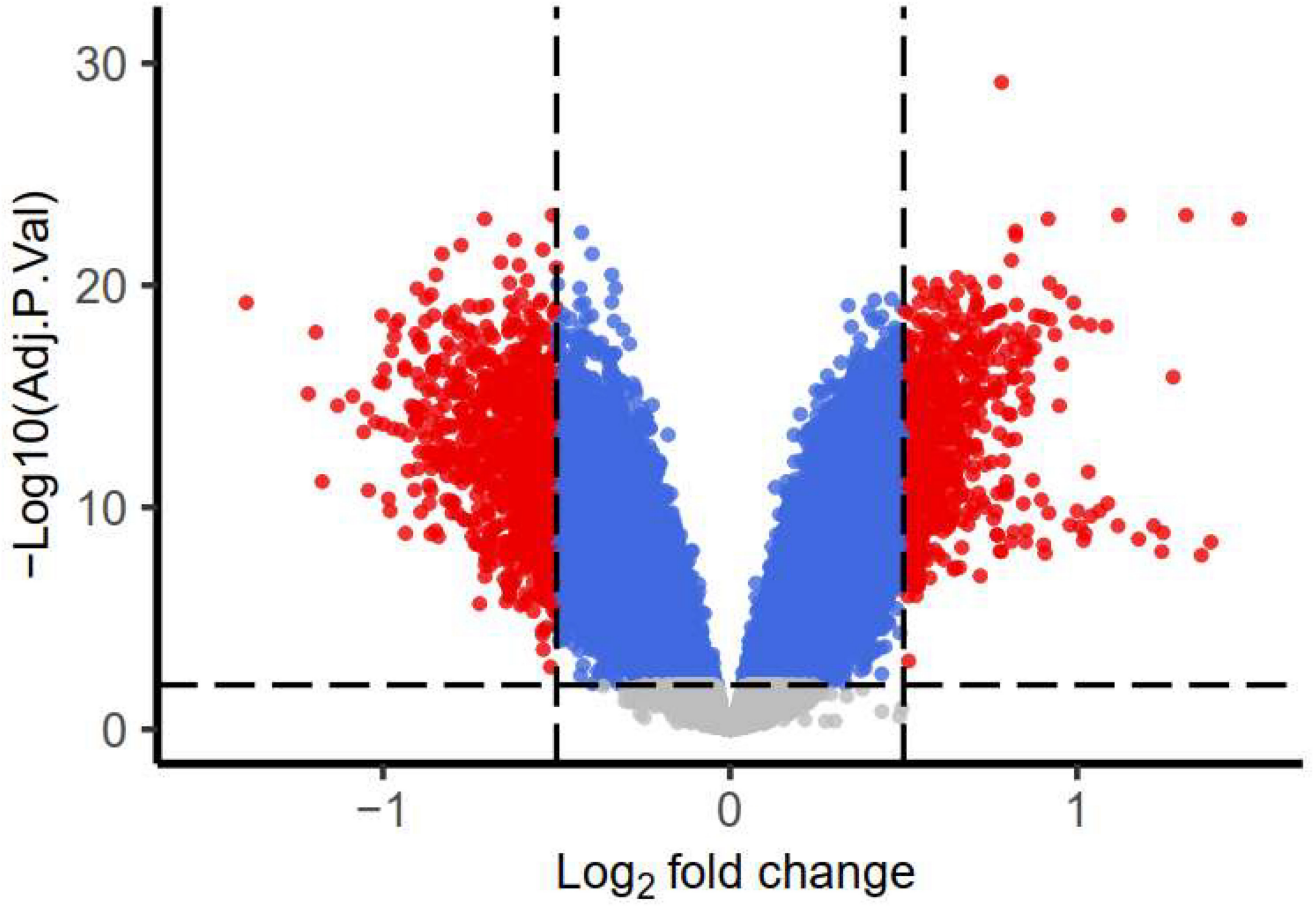
Volcano plot of mRNA expression in the blood of AD patients in comparison with healthy controls. This plot displays the distribution of transcripts according to their log2 (Fold Change) (*x*-axis) and statistical significance (-log10 adjusted *P*-value) (*y*-axis). The red dots display the significantly upregulated transcripts while the blue dots represent the significantly downregulated transcript. The positions of RPL21P28 and ATP2B1-AS1 among the candidates chosen for validation are shown.

A thorough differential expression analysis was primarily conducted on one of the greatest available public transcriptome datasets, GEO: GSE63060, including a total of 145 AD patients and 184 healthy individuals as the control group. In AD, a dispersion of the blood transcriptome landscape was noted. Hundreds of the transcripts matched our standards for differential expression overall (Adjusted *P*-value < 0.05, |log2 (Fold Change) | > 0.58). As shown in a volcano plot (Figure 1), several lncRNAs are among them. The two of the most statistically significant and reliable lncRNAs (the downregulated RPL21P28 and upregulated ATP2B1-AS1) were experimentally validated by using our independent clinical cohort.

### 3.2 Clinical cohort characteristics

A group of 25 AD patients and 25 healthy controls were included in the validation stage. As illustrated in Table 1; to prevent the interference of demographic variables in the expression analysis, the two groups were matched for sex (*P* = 0.785) and age (*P* = 0.671). The mean Mini-Mental State Examination (MMSE) score was notably lower in the AD patient group (18.2 ± 4.5) than the healthy controls (28.9 ± 1.1; *P* < 0.0001) as expected which confirmed the cognitive impairment in the patient group.

### 3.3 RT-qPCR validation confirms opposite dysregulation of ATP2B1-AS1 and RPL21P28 in AD plasma

Measurement of the relative expression levels of ATP2B1-AS1 and RPL21P28 in plasma samples using RT-qPCR validated the results obtained from the bioinformatic screening stage. The findings of the discovery analysis were strongly supported by the experimental validation.

**FIGURE 2:**
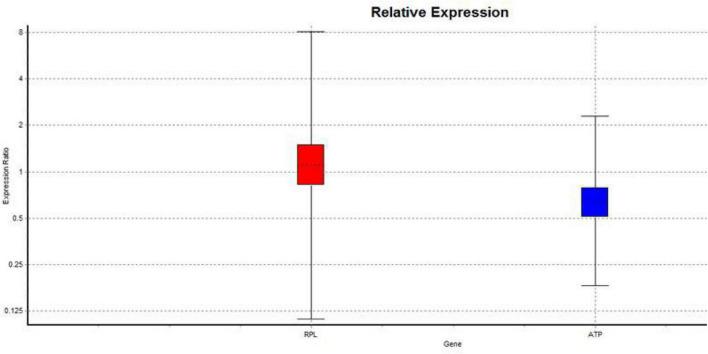
Relative expression level of lncRNAs in plasma of AD patients compared to healthy controls. Box-and-whisker plots display the relative expression of ATP2B1-AS1 and RPL21P28 as analyzed using RT-qPCR and normalized to endogenous control GAPDH by the 2^–ΔΔCT^ method. Data are presented for the AD (*n* = 25) and HC (*n* = 25) groups. Individual data points are also displayed on the graph. Median values are appointed by the horizontal line within the box. An unpaired *t*-test was used to ascertain the statistical significance (*P* < 0.001).

Specifically, plasma levels of ATP2B1-AS1 were significantly increased in AD patients compared with healthy controls (*P* < 0.001) (Figure 2). In contrast, RPL21P28 expression levels were significantly decreased in the plasma of AD patients (*P* < 0.001). These results are fully consistent with the expression patterns observed in the initial bioinformatic analysis (Figure 2).

### 3.4 High diagnostic accuracy of plasma ATP2B1-AS1 and RPL21P28 in AD

**FIGURE 3:**
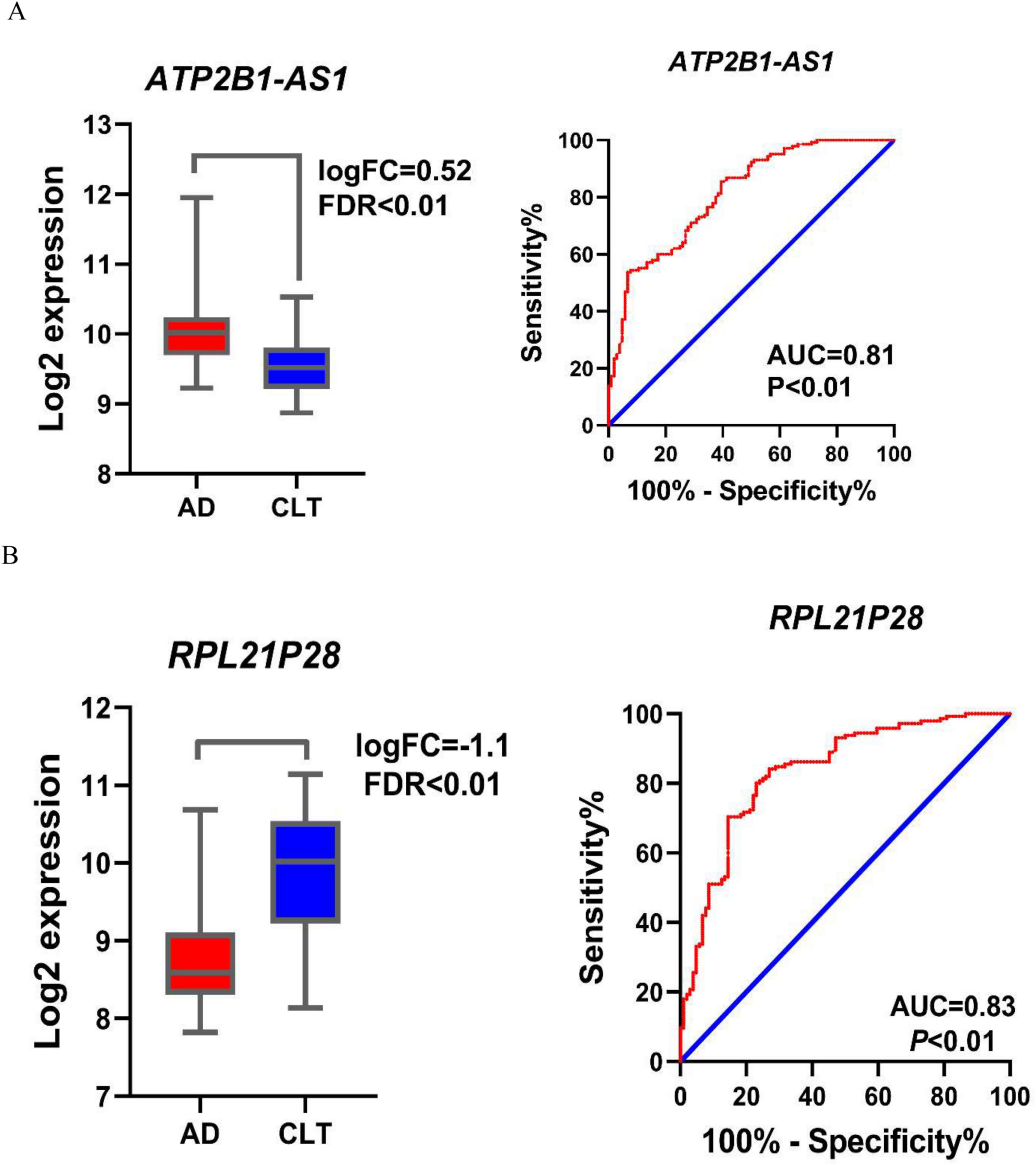
Diagnostic functioning of plasma lncRNAs in AD. Receiver operating characteristic (ROC) curves for **(A)** ATP2B1-AS1 and **(B)** RPL21P28. The AUC and the 95% CI for each lncRNA are given. The dashed line is a reference line representing chance discrimination (AUC = 0.5).

Then the suitability and qualification of the two lncRNAs as biomarkers for distinguishing AD patients from the healthy group were then evaluated using ROC curve analysis. The plasma lncRNAs showed robust potential in the diagnosis of AD. ATPB1-AS1 also showed an AUC of 0.81 (95% CI: 0.68–0.94) with 76% specificity and 84% sensitivity at an optimal cut-off value (Figure 3A). RPL21P28 showed a slightly higher diagnostic potential with AUC = 0.83 (95% CI: 0.71–0.95) and represented 72% specificity and 80% sensitivity (Figure 3B). Thus, both lncRNAs can be considered as suitable independent biomarkers for the diagnosis of AD.

### 3.5 The correlation between the expression levels of the lncRNA and the cognitive impairment severity in AD

**FIGURE 4:**
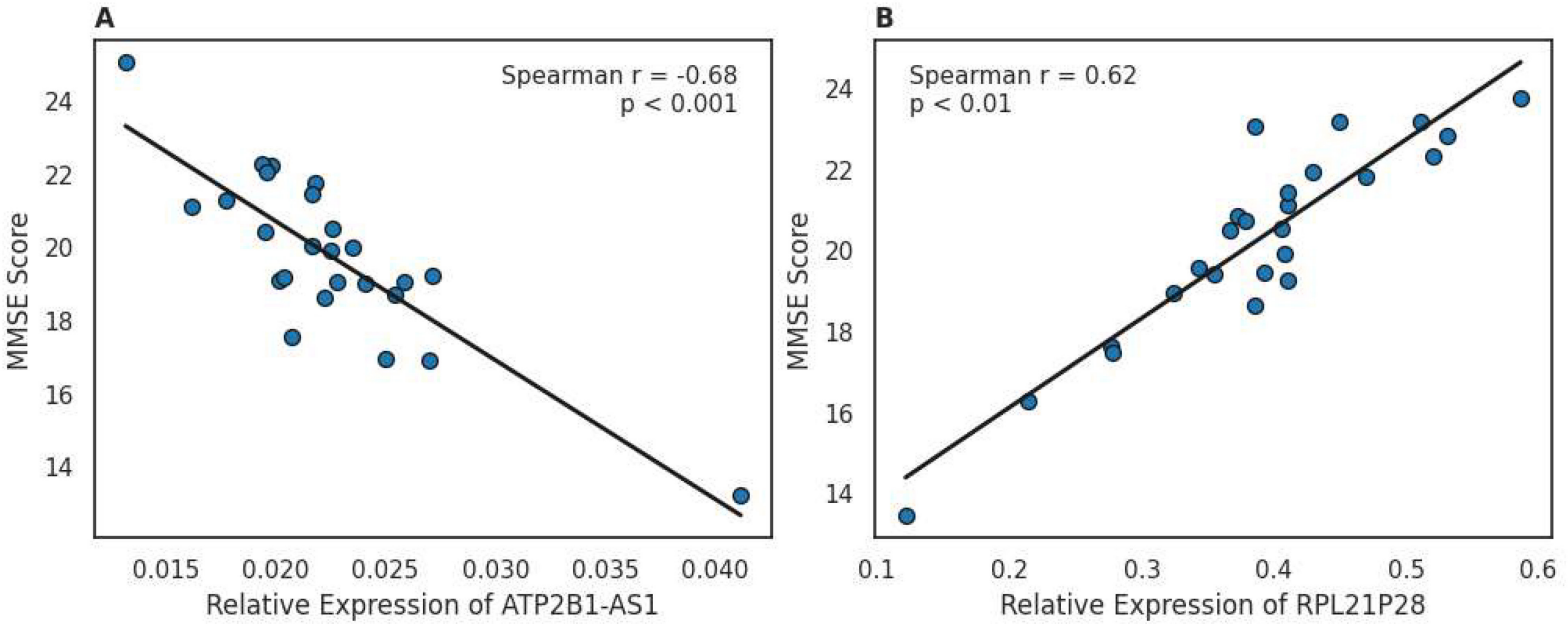
Relation between plasma lncRNAs and cognitive impairment severity in AD. Scatter diagrams illustrating the correlations between the relative expression of **(A)** ATP2B1-AS1 and **(B)** RPL21P28 with Mini-Mental State Examination scores in 25 AD patients. The Spearman rank order correlation coefficient (r) and *P*-value are also presented on individual plots. A solid line shows the line of best fit.

The MMSE scores showed a correlation which indicated that the plasma lncRNAs may be involved in AD severity. The relative expression of ATP2B1-AS1 displayed an inverse correlation to MMSE scores, with the Spearman correlation coefficient at −0.68 and *P*-value < 0.001 (Figure 4A). Similarly, based on *P*-value < 0.01 on the Spearman’s ranking correlation method (*r* = 0.62), MMSE scores and RPL21P28 expression appear to be positively correlated (Figure 4B). Basically, more severe cognitive impairments in AD patients may be associated with reduced plasma expression of RPL21P28 and increased plasma levels of ATP2B1-AS1.

### 3.6 The ceRNA network analysis predicts lncRNA regulation in neurodevelopmental and synaptic pathways

**FIGURE 5:**
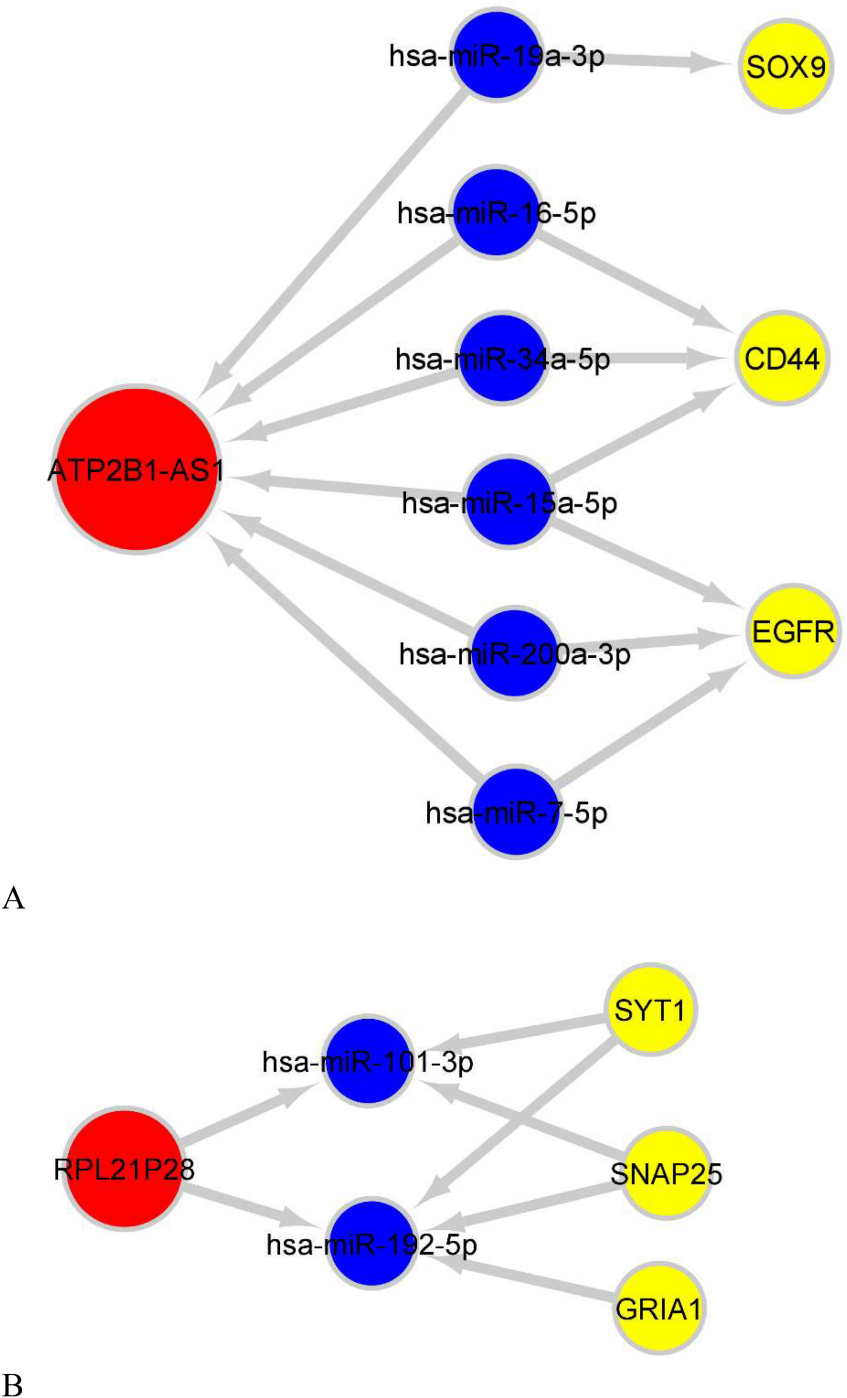
Predicted ceRNA regulatory networks for ATP2B1-AS1 and RPL21P28. Visualization of the constructed networks using Cytoscape. **(A)** The network centered on ATP2B1-AS1 (red octagon), representing its predicted interactions with key miRNAs (blue diamonds) and their downstream mRNA targets involved in neurodevelopment. **(B)** The network centered on RPL21P28, displaying its predicted interactions with miRNAs that target important synaptic function genes (yellow rectangles).

A precise tripartite ceRNA network was designed to investigate the possible biofunctions of ATP2B1-AS1 and RPL21P28. The two lncRNAs may therefore regulate the vital pathways involved in the pathogenesis of AD (Meftah, 2023). According to the ATP2B1-AS1 network prediction this lncRNA can function as a sponge for several miRNAs, such as hsa-miR-34a-5p and hsa-miR-15a-5p, which target key genes involved in neurodevelopment and cell survival like EGFR and SOX9 (Figure 5A). The analysis of the RPL21P28 network suggested that this lncRNA could competitively link to miRNAs like hsa-miR-101-3p which regulates the expression of core synaptic proteins such as SNAP25 and SYT1 (Figure 5B). This explains how AD synaptic dysfunction is linked to RPL21P28 downregulation.

## 4 Discussion

Establishing reliable non-invasive biomarkers for AD, has always been a remarkable challenge in modern medicine. In this study a unique two-lncRNA signature was identified in the plasma of AD patients by an accurate discovery-to-validation procedure. According to the results, the upregulation of ATP2B1-AS1 and downregulation of RPL21P28 distinguishes AD patients from the healthy group and also shows a meaningful correlation with the level of cognitive impairment. Moreover, according to the hypothesis proposed based on bioinformatic modeling in this study, these lncRNAs play a significant role in regulatory networks governing neurodevelopmental and synaptic pathways involved in AD pathogenesis.

Recent therapeutic advances in Alzheimer’s disease have increasingly focused on disease-modifying strategies targeting amyloid-β pathology. Monoclonal antibodies directed against aggregated amyloid-β, including aducanumab, lecanemab, and donanemab, have shown the ability to reduce amyloid plaque burden and modestly slow cognitive decline in patients with early AD in large randomized clinical trials (Mintun et al., 2021; Sevigny et al., 2016; van Dyck et al., 2023). These developments highlight the urgent need for accessible and minimally invasive biomarkers that can facilitate early diagnosis, identify individuals who may benefit from disease-modifying therapies, and monitor therapeutic response. Although current diagnostic frameworks frequently rely on cerebrospinal fluid analysis or amyloid/tau PET imaging, these approaches remain expensive and invasive for routine screening. Therefore, blood-based biomarkers are gaining considerable attention as practical alternatives. In this context, the lncRNA signature identified in the present study, consisting of the upregulated ATP2B1-AS1 and the downregulated RPL21P28, may represent a promising complementary biomarker panel for AD detection and potentially for monitoring disease progression or treatment response in future therapeutic settings.

The results of this research indicating the upregulation of ATP2B1-AS1 in AD patients are unprecedented in the field of neurodegeneration studies. Even though its function in the CNS has not yet been fully investigated, novel oncology studies indicated a relation between the elevated expression levels of ATP2B1-AS1 and proliferative and anti-apoptotic signaling, usually through ceRNA various mechanisms involving key miRNAs (Sarkar et al., 2019). Thus, according to the ceRNA network analysis in this study ATP2B1-AS1 can sponge away key miRNAs such as hsa-miR-15a-5p and hsa-miR-34a-5p. Both miRNAs, known as tumor suppressors, have a crucial role in neuronal homeostasis. For instance, it has been shown that hsa-miR-34a is upregulated in the AD brain, causing tau pathology and synaptic impairments (Sarkar et al., 2016). Increased expression of ATP2B1-AS1 prevents neurotoxic damages by preventing miRNA-mediated repression of pro-survival genes such as EGFR in a rather dysfunctional manner. Long-term maintenance of these pathways, however, may cause disturbances in cellular homeostasis which leads to the onset of the disease.

The results of our study indicate a significant downregulation of RPL21P28 and its expected relationship with synaptic function. Synaptic loss, also known as “synaptopathy,” has been identified in recent studies as one of the earliest and most important stages in the AD, preceding diffused neuronal loss, which is considered significantly related to cognitive impairment (Pei et al., 2020). Our ceRNA model identifies RPL21P28 as part of a regulatory network of key synaptic machinery genes, such as SNAP25 and SYT1. These genes encode the essential proteins of the SNARE complex, which is involved in neurotransmitter release and vesicle fusion (Wang et al., 2023). As observed in our AD group, reduced plasma expression of RPL21P28 can suppress miRNAs increased in AD, such as hsa-miR-101-3p, which target synaptic proteins (Brinkmalm et al., 2014). SNAP25 and SYT1 expression would then be suppressed, establishing a direct molecular connection between a peripheral biomarker and a significant pathogenic process in the brain of AD patients. These results strongly support the “window into the brain” theory, which states that free-floating ncRNAs in the blood represents contemporaneous central nervous system dysfunction.

The strong diagnostic role of the two-lncRNA signature is particularly important. With AUC values of over 0.80, our data propose that a minimally invasive plasma test measuring ATP2B1-AS1 and RPL21P28 levels may be a suitable screening and diagnostic technique in AD. This is especially important with the introduction of novel disease-modifying treatments, such as anti-amyloid antibodies, where early and accurate diagnosis is critical for both availability of treatment and the response. Considering the notable correlation between MMSE scores and these lncRNA levels these lncRNA can function as potential biomarkers for evaluating the clinical trial response or therapeutic effect. This shows the critical requirement for quantitative, objective assessment of disease activity that are more manageable and less troublesome than serial CSF taps or PET scans (Li et al., 2024).

Recent studies have increasingly highlighted the potential of circulating long non-coding RNAs (lncRNAs) as minimally invasive biomarkers for Alzheimer’s disease. Several blood-based lncRNAs have been reported to exhibit altered expression patterns in AD patients compared with cognitively normal individuals. For example, dysregulation of lncRNAs such as BACE1-AS, NEAT1, and MALAT1 has been associated with amyloidogenic processing, neuroinflammation, and neuronal dysfunction in AD pathogenesis (Fotuhi et al., 2022; Zhang et al., 2017). Moreover, emerging transcriptomic studies using peripheral blood samples have suggested that circulating lncRNA signatures may provide valuable diagnostic information and may complement established protein biomarkers such as amyloid-β and phosphorylated tau (Zhao et al., 2021).

These findings support the growing concept that blood-derived lncRNAs could serve as accessible molecular indicators of neurodegenerative processes. In line with these observations, the two-lncRNA signature identified in the present study further expands the repertoire of candidate blood-based RNA biomarkers for AD and highlights the potential regulatory involvement of lncRNA-mediated networks in disease development.

Despite the validity of our findings, several limitations should be acknowledged. First, the validation cohort included a relatively limited number of participants (*n* = 25 per group). Although statistically significant differences were observed for the pre-selected lncRNA candidates, this modest sample size may reduce the statistical power to detect smaller effects and may limit the generalizability of the findings. Therefore, larger, multicenter studies involving ethnically diverse populations are required to confirm the robustness and external validity of the proposed lncRNA signature.

Second, although the diagnosis of Alzheimer’s disease (AD) in this study was established by experienced neurologists according to the National Institute on Aging-Alzheimer’s Association (NIA-AA) diagnostic framework and supported by structural MRI findings, the absence of cerebrospinal fluid (CSF) biomarkers or amyloid/tau PET imaging in our cohort limits direct comparison with the current biological definition of AD and gold-standard biomarker profiles. In addition, the single-center recruitment strategy may introduce potential selection bias and may further restrict the generalizability of our results.

Third, the present study employed a cross-sectional design. While this design is appropriate for identifying associations between circulating lncRNAs and AD, it does not allow assessment of temporal dynamics or causal relationships. Consequently, longitudinal investigations are necessary to determine whether these circulating lncRNA alterations precede measurable cognitive decline and whether they may serve as early predictors of disease progression or conversion from mild cognitive impairment (MCI) to Alzheimer’s disease.

Fourth, the ceRNA regulatory network proposed in this study was constructed primarily using bioinformatic predictions derived from publicly available interaction databases and network analysis platforms. Although such computational approaches are widely used to explore lncRNA-mediated regulatory mechanisms and are supported by growing evidence highlighting the importance of lncRNAs and ceRNA interactions in Alzheimer’s disease pathogenesis, the predicted interactions should be interpreted cautiously. Direct experimental validation of key regulatory relationships—such as the interaction between RPL21P28 and hsa-miR-101-3p—using functional assays (e.g., luciferase reporter assays, RNA pull-down experiments, or knockdown/overexpression studies) will be necessary to confirm the biological relevance of the proposed regulatory axis.

Finally, we did not directly compare the diagnostic performance of the identified lncRNA signature with established blood-based biomarkers of AD, including plasma amyloid-β (Aβ), phosphorylated tau, and neurofilament light chain. As a result, the potential incremental diagnostic value of these circulating lncRNAs relative to currently emerging biomarker platforms could not be assessed. Future studies integrating lncRNA biomarkers with established molecular markers and including additional neurodegenerative disease control groups (e.g., Parkinson’s disease or frontotemporal dementia) would be valuable to further evaluate the specificity, sensitivity, and clinical utility of this biomarker panel.

## 5 Conclusion

The current study excellently confirmed a new and unique plasma-derived lncRNA signature (including the downregulated RPL21P28 and the upregulated ATP2B1-AS1), that effectively distinguishes AD patients from healthy controls and correlates with the degree of their cognitive impairments. The bioinformatic and laboratory data of this study demonstrated that these lncRNAs are not just byproducts of the disease, but y rather are functional components of regulatory networks that control the key synaptic and neurodevelopmental prominent pathways in AD. These results introduced a minimally invasive blood test for AD and provide new opportunities for exploring lncRNA-targeted therapy by restoration of synaptic integrity and neuronal homeostasis in AD.

## Data Availability

The datasets used in this study are publicly available in the NCBI Gene Expression Omnibus (GEO) repository under accession numbers GSE4757, GSE5281, GSE28146, and GSE48350. These data can be accessed directly via the GEO repository at https://www.ncbi.nlm.nih.gov/geo/.
